# Geographic Variations in Incremental Costs of Heart Disease Among Medicare Beneficiaries, by Type of Service, 2012

**DOI:** 10.5888/pcd13.160209

**Published:** 2016-12-29

**Authors:** Rita Wakim, Matthew Ritchey, Jason Hockenberry, Michele Casper

**Affiliations:** 1 Division for Heart Disease and Stroke Prevention, National Center for Chronic Disease Prevention and Health Promotion, Centers for Disease Control and Prevention, Atlanta, Georgia; 2 Department of Health Policy and Management, Rollins School of Public Health, Emory University, Atlanta, Georgia.

## Abstract

Using 2012 data on fee-for-service Medicare claims, we documented regional and county variation in incremental standardized costs of heart disease (ie, comparing costs between beneficiaries with heart disease and beneficiaries without heart disease) by type of service (eg, inpatient, outpatient, post-acute care). Absolute incremental total costs varied by region. Although the largest absolute incremental total costs of heart disease were concentrated in southern and Appalachian counties, geographic patterns of costs varied by type of service. These data can be used to inform development of policies and payment models that address the observed geographic disparities.

## Objective

Treatment of heart disease is one of the largest drivers of health care costs in the United States, accounting for approximately $273 billion annually (1). Although geographic variation in Medicare costs is well documented (2), little is known about geographic variation in costs for beneficiaries with heart disease. The objective of this study was to document geographic variation in incremental health care costs among fee-for-service Medicare beneficiaries diagnosed with heart disease and costs among beneficiaries without heart disease by region, county, and type of service.

## Methods

We used 2012 data from the Centers for Medicare & Medicaid Services’ (CMS) Chronic Conditions Data Warehouse, a database with 100% of fee-for-service Medicare enrollment and claims data (3). We limited the data to Medicare beneficiaries aged 65 years or older. We excluded Medicare beneficiaries enrolled in Medicare Advantage or in Medicare Part A only or Part B only. Beneficiaries with diagnosed heart disease were identified according to the following CMS Hierarchical Condition Category codes: congestive heart failure (code 80), acute myocardial infarction (code 81), unstable angina/acute ischemic heart disease (code 82), and specified heart arrhythmias (code 92). Hierarchical Condition Category codes were created by CMS to predict patient costs and map diagnostic codes into diagnostic groupings (4). All major ICD-9-CM (*International Classification of Diseases, Ninth Revision, Clinical Modification*) codes for heart disease are included in the HCC except codes 390–398 (acute and chronic rheumatic fever). We calculated total costs by summing Medicare payments, beneficiary payments, and third-party payments for the following service categories: inpatient, outpatient, post-acute care, hospice, physician, procedure/imaging, and other. Because calculating costs incurred directly for heart disease care is not possible, we calculated incremental costs incurred among beneficiaries with heart disease above and beyond the costs incurred by beneficiaries without heart disease. Incremental costs were calculated as the difference between mean annual costs per capita for beneficiaries with heart disease and beneficiaries without heart disease in 2 ways: 1) as the absolute difference, by subtracting the cost per beneficiary without heart disease from the cost per beneficiary with heart disease, and 2) as the relative difference, by dividing the cost per beneficiary with heart disease by the cost per beneficiary without heart disease. Incremental costs were age-standardized (2000 standard US population age 65 years or older) and analyzed by region, county, and type of service. We generated county-level maps of the prevalence of heart disease and the age-standardized absolute incremental values for total costs, inpatient costs, and outpatient costs of heart disease. Subnational cost analyses were adjusted to eliminate geographic differences in payment rates (eg, local wages, input prices) (5). We used SAS version 9.4 (SAS Institute Inc) for the analyses.

## Results

Total costs among beneficiaries with heart disease was approximately $96 billion in 2012. The absolute and relative incremental total costs of heart disease per beneficiary were $10,345 and 2.6 per capita, respectively. Inpatient, post-acute care, and outpatient services accounted for the vast proportion of total absolute incremental costs (37%, 25%, and 18%, respectively). Relative incremental costs of heart disease varied minimally by region (ranges, 2.5–2.7 for total costs and 1.5–3.0 per service type) (Table). However, absolute incremental total costs varied by region, and the pattern of variation was similar to that of heart disease prevalence (Figure). The highest mean absolute incremental total costs were in the South ($11,581 per capita). The highest mean incremental inpatient costs were also in the South ($4,101 per capita), but the highest mean incremental outpatient costs were in the Midwest ($2,225 per capita). This pattern was further elucidated at the county level. Counties with incremental total costs in the highest quintile (range, $12,443–$28,001) were concentrated primarily in the South and parts of Appalachia (Map A). The highest incremental inpatient costs (highest quintile range, $4,648–$19,329) were in counties in the South and Northeast, including Appalachia (Map C), whereas the highest incremental outpatient costs (top quintile range, $2,363–$7,798) were concentrated primarily in counties in the West and Midwest (Map D).

**Table:** Age-Standardized^a^ Prevalence of Heart Disease and Mean Costs per Fee-for-Service Medicare Beneficiary With Heart Disease^b^, Compared With Costs for Beneficiary Without Heart Disease, by US Census Region^c^, 2012^d^

Census Region^c^	Age-Standardized^a^ Prevalence of Heart Disease^b^, %	Age-Standardized^a^ Mean Cost per Beneficiary With Heart Disease^b^, $ (% of Total)	Age-Standardized^a^ Mean Cost per Beneficiary Without Heart Disease^b^, $ (% of Total)	Mean Absolute Incremental Cost^e^ per Beneficiary With Heart Disease^b^, $ (% of Total)	Mean Relative Incremental Cost^f^ of Heart Disease^b^
**Total (3,122 counties)**					
Total	20.4	16,986 (100)	6,641 (100)	10,345 (100)	2.6
Inpatient		5,972 (35)	2,187 (33)	3,785 (37)	2.7
Post-acute care		4,037 (24)	1,427 (21)	2,610 (25)	2.8
Outpatient		2,966 (17)	1,081 (16)	1,885 (18)	2.7
Physician		1,751 (11)	838 (13)	913 (9)	2.1
Hospice		1,057 (6)	356 (5)	701 (7)	3.0
Procedure/imaging		1,016 (6)	665 (11)	351 (3)	1.5
Other		187 (1)	87 (1)	100 (1)	2.1
**Northeast (217 counties)**					
Total	20.5	16,848 (100)	6,569 (100)	10,279 (100)	2.6
Inpatient		6,100 (37)	2,098 (32)	4,002 (39)	2.9
Post-acute care		3,732 (22)	1,311 (20)	2,421 (24)	2.8
Outpatient		2,904 (17)	1,134 (17)	1,770 (17)	2.6
Physician		2,053 (12)	985 (15)	1,068 (10)	2.1
Hospice		818 (5)	272 (4)	546 (5)	3.0
Procedure/imaging		1,066 (6)	692 (11)	374 (4)	1.5
Other		175 (1)	77 (1)	98 (1)	2.3
**Midwest (1,053 counties)**					
Total	19.5	16,129 (100)	6,360 (100)	9,769 (100)	2.5
Inpatient		5,852 (37)	2,183 (34)	3,669 (38)	2.7
Post-acute care		3,612 (22)	1,274 (20)	2,338 (24)	2.8
Outpatient		3,499 (22)	1,274 (20)	2,225 (22)	2.7
Physician		1,522 (9)	725 (11)	797 (8)	2.1
Hospice		543 (3)	179 (3)	364 (4)	3.0
Procedure/imaging		922 (6)	626 (10)	296 (3)	1.5
Other		179 (1)	99 (2)	80 (1)	1.8
**South (1,417 counties)**					
Total	20.1	18,948 (100)	7,367 (100)	11,581 (100)	2.6
Inpatient		6,421 (34)	2,320 (31)	4,101 (35)	2.8
Post-acute care		4,979 (26)	1,755 (24)	3,224 (28)	2.8
Outpatient		2,659 (14)	980 (13)	1,679 (14)	2.7
Physician		1,962 (10)	945 (13)	1,017 (9)	2.1
Hospice		1,660 (9)	556 (8)	1,104 (10)	3.0
Procedure/imaging		1,089 (6)	712 (10)	377 (3)	1.5
Other		178 (1)	99 (1)	79 (1)	1.8
**West (435 counties) **					
Total	17.8	13,953 (100)	5,535 (100)	8,418 (100)	2.5
Inpatient		4,993 (36)	1,913 (35)	3,080 (37)	2.6
Post-acute care		2,791 (20)	976 (18)	1,815 (22)	2.9
Outpatient		3,032 (22)	1,033 (18)	1,999 (23)	2.9
Physician		1,440 (10)	682 (12)	758 (9)	2.1
Hospice		552 (4)	194 (4)	358 (4)	2.8
Procedure/imaging		968 (7)	641 (12)	327 (4)	1.5
Other		177 (1)	96 (1)	81 (1)	1.8

a 2000 Standard US population age 65 years or older.

b Identified according to the following Centers for Medicare & Medicaid Services’ Hierarchical Condition Category codes: congestive heart failure (code 80), acute myocardial infarction (code 81), unstable angina/acute ischemic heart disease (code 82), and specified heart arrhythmias (code 92).

c Northeast includes Connecticut, Maine, Massachusetts, New Hampshire, New Jersey, New York, Pennsylvania, Rhode Island, and Vermont. Midwest includes Illinois, Indiana, Iowa, Kansas, Michigan, Minnesota, Missouri, Nebraska, North Dakota, Ohio, South Dakota, and Wisconsin. South includes Alabama, Arkansas, Delaware, District of Columbia, Florida, Georgia, Kentucky, Louisiana, Maryland, Mississippi, North Carolina, Oklahoma, South Carolina, Tennessee, Texas, Virginia, and West Virginia. West includes Alaska, Arizona, California, Colorado, Hawaii, Idaho, Montana, Nevada, New Mexico, Oregon, Utah, Washington, and Wyoming.

d Data from Centers for Medicare & Medicaid Services’ Chronic Conditions Data Warehouse (3) limited to Medicare beneficiaries aged 65 years or older; beneficiaries enrolled in Medicare Advantage or in Medicare Part A only or Part B only were excluded.

e Calculated by subtracting the cost per beneficiary without heart disease from the cost per beneficiary with heart disease.

f Calculated by dividing the age-standardized mean cost per beneficiary with heart disease by the age-standardized mean cost per beneficiary without heart disease.

**Figure:**
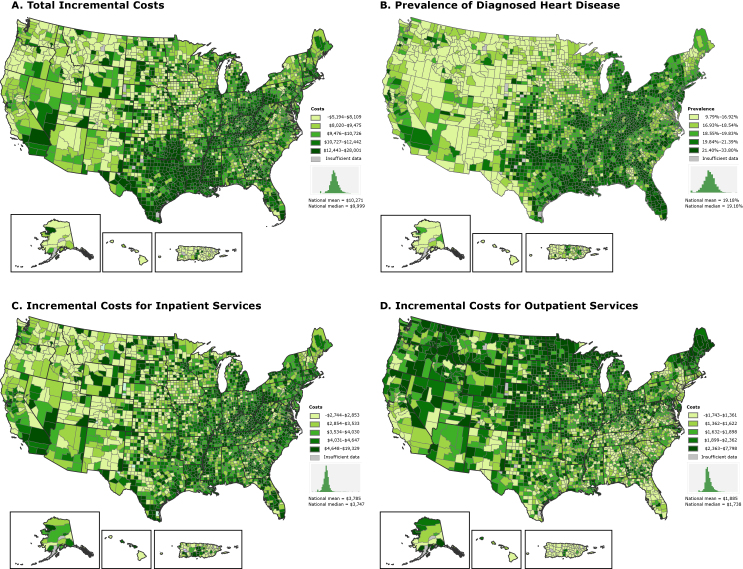
Incremental costs of care and prevalence of diagnosed heart disease for fee-for-service Medicare beneficiaries by county, 2012. A. Total incremental costs of care. Seven counties had negative incremental values. B. Prevalence of diagnosed heart disease. C. Incremental costs for inpatient services. Nineteen counties had negative incremental values. D. Incremental costs for outpatient services. Ten counties had negative incremental values. All counties with negative incremental values had fewer than 30 beneficiaries with heart disease. Beneficiaries with diagnosed heart disease were identified according to the following Centers for Medicare & Medicaid Services Hierarchical Conditions Category codes: congestive heart failure (80), acute myocardial infarction (81), unstable angina/acute ischemic heart disease (82), and specified heart arrhythmias (92). Incremental costs were calculated as the difference between the mean annual costs per capita for beneficiaries with heart disease and the mean annual costs per capita for beneficiaries without heart disease. For all maps, the category “Insufficient data” indicates that data from counties with fewer than 10 Medicare beneficiaries were suppressed. Data source: Centers for Medicare & Medicaid Services (5). We used ESRI’s ArcGIS 10.3 software to produce the maps.

## Discussion

We found substantial absolute incremental costs of heart disease among fee-for-service Medicare beneficiaries. Inpatient, post-acute care, and outpatient services contributed most to incremental costs. Although the highest absolute incremental total costs of heart disease were concentrated in counties in the Southeast and Appalachia, geographic patterns of cost varied by type of service. For example, counties in the South and southern Appalachia generally had the highest absolute incremental inpatient costs but the lowest absolute incremental outpatient costs.

Researchers disagree on which factors drive the regional variation in health care costs among Medicare beneficiaries. Among these factors are the need for medical care (eg, determined by the prevalence or severity of disease or prevalence of comorbid conditions) and the provision of care (6,7). Our findings expand on previous research by describing geographic variation in incremental costs of heart disease and offer initial insights into how differences in the use of services might drive some of this variation. Regions with the highest total incremental costs had the highest heart disease prevalence and highest incremental inpatient costs. At first glance, these patterns suggest that these regions may have had a higher prevalence of severe heart disease or heart disease–related comorbidities, which necessitated increased use of higher-cost, higher-acuity care. However, regions with the highest total incremental costs tended to have the lowest outpatient incremental costs and the lowest overall outpatient costs, patterns not necessarily congruent with managing a population with greater health care needs. Perhaps practice patterns in these regions lead to substituting more costly inpatient services for outpatient services.

One limitation of this study was the use of claims data rather than clinical data to identify beneficiaries with heart disease. However, claims data are reliable in assessing cardiovascular outcomes (8). Also, standardized payments do not adjust for health status or several sociodemographic factors; these factors may contribute to differences in service use.

Geographic variation in the incremental costs of care for beneficiaries with heart disease and geographic differences in use of services suggest a non-uniform use of resources to prevent and treat heart disease. Public health, health care systems, and health plans can use these findings to inform development of policies and payment models that address geographic disparities in service use and overall costs for care among Medicare beneficiaries with heart disease.
